# Sitagliptin Reduces Cardiac Apoptosis, Hypertrophy and Fibrosis Primarily by Insulin-Dependent Mechanisms in Experimental type-II Diabetes. Potential Roles of GLP-1 Isoforms

**DOI:** 10.1371/journal.pone.0078330

**Published:** 2013-10-21

**Authors:** Belén Picatoste, Elisa Ramírez, Alicia Caro-Vadillo, Cristian Iborra, Jesús Egido, José Tuñón, Óscar Lorenzo

**Affiliations:** 1 Instituto de Investigaciones Sanitarias-Fundación Jiménez Díaz, Madrid, Spain; 2 Veterinary School, Complutense University, Madrid, Spain; Bristol Heart Institute, University of Bristol, United Kingdom

## Abstract

**Background:**

Myocardial fibrosis is a key process in diabetic cardiomyopathy. However, their underlying mechanisms have not been elucidated, leading to a lack of therapy. The glucagon-like peptide-1 (GLP-1) enhancer, sitagliptin, reduces hyperglycemia but may also trigger direct effects on the heart.

**Methods:**

Goto-Kakizaki (GK) rats developed type-II diabetes and received sitagliptin, an anti-hyperglycemic drug (metformin) or vehicle (n=10, each). After cardiac structure and function assessment, plasma and left ventricles were isolated for biochemical studies. Cultured cardiomyocytes and fibroblasts were used for *in* *vitro* assays.

**Results:**

Untreated GK rats exhibited hyperglycemia, hyperlipidemia, plasma GLP-1 decrease, and cardiac cell-death, hypertrophy, fibrosis and prolonged deceleration time. Moreover, cardiac pro-apoptotic/necrotic, hypertrophic and fibrotic factors were up-regulated. Importantly, both sitagliptin and metformin lessened all these parameters. In cultured cardiomyocytes and cardiac fibroblasts, high-concentration of palmitate or glucose induced cell-death, hypertrophy and fibrosis. Interestingly, GLP-1 and its insulinotropic-inactive metabolite, GLP-1(9-36), alleviated these responses. In addition, despite a specific GLP-1 receptor was only detected in cardiomyocytes, GLP-1 isoforms attenuated the pro-fibrotic expression in cardiomyocytes and fibroblasts. In addition, GLP-1 receptor signalling may be linked to PPARδ activation, and metformin may also exhibit anti-apoptotic/necrotic and anti-fibrotic direct effects in cardiac cells.

**Conclusions:**

Sitagliptin, via GLP-1 stabilization, promoted cardioprotection in type-II diabetic hearts primarily by limiting hyperglycemia e hyperlipidemia. However, GLP-1 and GLP-1(9-36) promoted survival and anti-hypertrophic/fibrotic effects on cultured cardiac cells, suggesting cell-autonomous cardioprotective actions.

## Introduction

The global prevalence of type-II diabetes mellitus (T2DM) has increased in such a way that has achieved epidemic proportions [1]. Experimental and clinical studies have shown an association between T2DM and cardiomyopathy, being defined by functional and structural changes at the level of myocardium, independent of any vascular or cardiac diseases. Diabetic cardiomyopathy (DCM) is characterized by myocardial apoptosis, hypertrophy and subsequent fibrosis, as well as cardiac dysfunction [2]. The intervention to prevent the development of fibrosis has been proposed for the treatment of DCM [3, 4]. An excess of plasma free fatty-acid (FFA) and glucose triggers pro-fibrotic factors and extracellular matrix (ECM) deposition from fibroblasts and myocytes [2,3]. However, the molecular underlying mechanisms of these responses are poorly known, leading to a lack of treatment. In this regard, metformin is the only anti-diabetic conclusively probed to avoid cardiac complications in diabetes. Unfortunately, this insulin-sensitizer can produce unwanted side effects [1]. Dipeptidyl peptidase-IV (DPP-IV) inhibitors, as sitagliptin, are a new class of anti-diabetics that prevent the degradation of insulinotropic incretins, without producing hypoglycemia [5]. The most active incretin is glucagon-like peptide-1-(7-36), usually termed GLP-1. GLP-1 is released from intestinal L-cells to the circulation in response to ingested nutrient. GLP-1 regulates blood glucose mainly by enhancing pancreatic insulin secretion, and confers cardioprotection after myocardial infarction, congestive heart failure and ischemia [6,7]. However, the expression of DPP-IV and GLP-1 receptors (GLP-1R) has been also described in different tissues including liver, vessel and heart, suggesting extra-pancreatic actions [6-9]. Via RISK (cAMPPKAPI3KAkt) pathway GLP-1R induce transcription factor activation [10,11]. In this sense, peroxisome proliferator-activated receptors (PPAR) are FFA-binding nuclear receptors that act as transcription factors to regulate cardiac metabolic and inflammatory genes [12]. In particular, PPARδ may control pro-fibrotic genes to prevent cardiac fibrosis and heart failure [13]. However, due to the plasma DPP-IV activity, GLP-1 is degraded within minutes to the insulinotropic-inactive GLP-1(9-36) [5]. The aim of this study was to investigate whether sitagliptin could induce cardioprotection for T2DM hearts by GLP-1/PPARδ direct actions on cardiac cells.

## Methods

### Ethics Statement

These investigations adhered to the Guide for the Care and Use of Laboratory Animals (NIH Publication No. 85–23, revised 1996) and the Ethics Committee of the IIS-Fundación Jiménez Díaz Hospital granted approval for these experiments (ref. 2012/5). 

### Animal Model

A polygenic non-obese non-hypertensive model of T2DM was used for the study. Male Goto-Kakizaki (GK) rats were purchased from Taconic, Denmark and were kept on an artificial 12-hour light-dark cycle (7 a.m.-7 p.m.) at 25°C. GK exhibit similar metabolic, hormonal and vascular disorders that the human T2DM, offering a convenient model for the study of T2DM per se, without the confounding effects of obesity or hypertension [14]. Once GK became diabetic (at 16th week), some were treated with sitagliptin [Merck Sharp & Dohme (Spain), 10 mg/Kg/day] or metformin clorhidrato [Acofarma (Spain), 200 mg/Kg/day]. Both drugs were dissolved in water and daily administrated (10 a.m.) by a gavage. Vehicle-treated GK and wistar were also examined. N=10, each group. Body weight, diet consumption and systolic blood pressure (measured by tail-cuff method) were weekly evaluated. After 10 weeks of treatment, plasma (collected from cava vein) and hearts were isolated (3-7 p.m.) under isoflurane (1.5% in O_2_) anaesthesia. Plasma lipid profile, glucose, hepatic enzymes and renal parameters were enzymatically measured in the clinical department of the Hospital. Hearts were rinsed, dried and weighted. After atria excision, a ventricular slice was included in p-formaldehyde and paraffin for histology. Then, left ventricles were frozen in liquid-N2 for biochemical assays. 

### Glucose tolerance test

Glucose tolerance was evaluated as published elsewhere [15]. Blood samples were collected (from tail vein) at the day before sacrifice after overnight fasting (N=10, each group). Then, rats received the corresponding dose of vehicle or sitagliptin and plasma was immediately obtained, after which glucose solution (0.5 g/kg) was administrated by i.p. Fifteen and sixty minutes after glucose loading, plasma was obtained again. Plasma glucose and insulin were measured by ELISA kits (Mercodia AB; Sweden). Plasma GLP-1 was determined by modification of Orskov method [16]. Samples [collected in glass tubes with DPP-IV inhibitors (Vacutainer P700, BD; USA)] were mixed with 0.5M EDTA, 10,000 UIC/ml aprotinin and absolute ethanol for 1h at 4°C, and centrifuged (3,000 rpm, 15 min at 4°C). Supernatants were frozen in liquid-N_2_, lyophilized and dissolved in 0.2M glycin-0.5% human serum albumin-500U/ml aprotinin solution (pH 8,8). Then, 100 µl were used for GLP-1 quantification by ELISA (Epitope Diagnostic Inc.; USA). 

### Cardiac structure and function measurement

Cardiac echocardiography was performed under 1.5% isoflurane-O_2_ anaesthesia in all rats before (not shown) and after the treatments. Both M-mode and two-dimensional (2D) echocardiograms were obtained using a 12 MHz ultra-band sector transducer (En Visor-C-HD, Philips). Images were obtained from the left and right parasternal window in a supine decubitus position. The following parameters were measured and calculated from M-mode tracing: left ventricular (LV) end-diastolic diameter (LVDD), LV end-systolic diameter (LVSD) and ejection fraction (EF; by Teichzol method). Wall thickness of four segments [anterior, inter-ventricular-septum (IVS), lateral, and posterior (LVPW) walls] was evaluated on short axis 2D images. LV mass index (LVMI) was calculated according to Devereux method using LVPW, IVS and LVDD parameters, and normalized to body weight as previously described [17]. For histological quantification of the LVPW and IVS thicknesses, serial paraffin sections (4 µm) of half-height sliced myocardium were fixed on slides and stained with Haematoxylin/Eosin (H/E). LVPW and IVS thicknesses were evaluated as mean of 4-5 measurements in the same heart region of all rats by using Metamorph software. A representative photograph taken with an optical microscopy, and the score for each rat are shown. Cell size of LVPW cardiomyocytes was quantified in 50 transversally oriented H/E-stained cells of twenty randomly fields by average of the cross-sectional areas (at nucleus level), using Metamorph. 

### Cardiac fibrosis and cell-death examination

Masson trichrome was used to detect extra-cellular matrix (ECM) deposition by sequent addition of Bouin’s, Weigert’s and Biebrich solutions (Bio-Optica, Milan, Italy) on paraffin sections (4 µm) of all myocardia. Interstitial, perivascular and replacement fibrosis were quantified together on five fields of each myocardium using the Metamorph software. Photographs with a scale bar were taken at 40x magnification under optical microscopy. Apoptosis was detected by a TUNEL-based apoptosis detection kit, following manufacture’s instructions (ApopTag®, Invitrogen). The percentage of TUNEL-positive nuclei relative to total nuclei was determined in a blinded manner by counting 200-300 cells on ten randomly chosen fields per coverslip for each myocardium. *In vitro*, cells were cultured in chamber slides (Nunc; Naperville, IL), stimulated, fixed with methanol:acetone (1:1) and nuclear-stained with DAPI. Condensed, pyknotic and/or fragmented nuclei of death cells were identified by confocal microscopy and were counted by using Metamorph. Necrosis was evaluated in the hearts by loss of cytoskeleton vinculin in paraffin-fixed slides by incubations with an anti-vinculin antibody followed by a biotinylated secondary antibody (Cell Signalling, USA), as previously described [32]. Representative photographs were taken under confocal microscopy. In cultured cardiomyocytes necrosis was measured by the release of the cytolytic glucose 6-phosphate dehydrogenase (G6PD) from damage and dying cells into the media, using the Vybranto Cytotoxicity Assay Kit (Invitrogen). 

### Cultured cardiomyocytes and fibroblasts

A derived cardiac muscle cell line, designated HL-1, from the AT-1 mouse atrial cardiomyocyte tumour lineage was used for *in vitro* assays. These cells (kindly given by Dr. Zalba, Pamplona, Spain) [18] retain differentiated cardiac morphological, biochemical, and electrophysiological properties, and exhibit a pattern of gene expression similar to that of adult myocytes [19]. HL-1 were grown in gelatin/fibronectin-coated plates with Claycomb medium (Sigma-Aldrich; USA) supplemented with 10% (vol/vol) heat-inactivated foetal calf serum (FBS), 10 μM norepinephrine, 100 IE/ml Na^+^-penicillin, 2 mM L-glutamine and 5 mM D-glucose (Sigma-Aldrich; USA). Cardiac fibroblasts from adult male wistar rats were obtained by differential centrifugation of cardiac cells released after retrograde Langendorff perfusion with a Ca^++^-free tyrode solution and enzymatic digestion with 250 UI of collagenase type-II, as previously described [20]. The fibroblasts were resuspended in DMEM medium supplemented with 10% FBS, 10 mM L-glutamine, 100 U/ml penicillin/streptomycin, 10 mM L-pyruvate and 2 mM HEPES. Cells were used at 2-3 passages. Also, a cell line of interstitial fibroblasts (TFB; kindly given by Dr. Nielson, USA) [21] derived from murine kidney was growth in RPMI-1640 medium supplemented with 5% FBS, 2% penicillin/streptomycin and 5 mM glucose (Sigma). All cells were switched to serum-free quiescent medium overnight before stimulation. The hyperlipidemic or hyperglycemic conditions were mimicked by incubation (6-24h) with high concentration of a common saturated FFA (HF; sodium palmitate 16:0, 0.25 mM) or glucose (HG; D-glucose 33 mM) (Sigma), respectively. Palmitate was previously conjugated with BSA in a 3:1 molar ratio as published elsewhere [22]. In control cells, BSA was added as described but in the absence of palmitate. Some cells were pre-treated with sitagliptin (1h, 0.5 μM) since its peak plasma concentration after a single oral dose of 100 mg (10 mg in rats) is about 0.5-0.6 μM, which is reported to produce nearly complete inhibition of DPP-IV [23]. GLP-1(7-36) (1 nM), GLP-1(9-36) (0.3 nM) (Sigma) or metformin clorhidrato (5 mM) were added 30 min before stimulation. A PPARδ-agonist [GW0742 (10 μM), Sta. Cruz Biotech, USA] or antagonist [G5797 (10 μM), Sigma] was added 24h before stimulation. 

### Western Blot (WB)

A piece (50 mg) of homogenized ventricle (by Bullet Blender, Cultek) or cell extract were dissolved in protein lysis buffer (50 mM Tris-HCl pH 7.5, 1 mM EDTA, 2% SDS + 1/250 mammalian protease inhibitors), and equal amounts (20-30 μg) of protein extracts were separated on polyacrylamide gels, transferred to membranes and probed with specific primary antibodies [anti-fibronectin (Millipore, Darmstadt, Germany), -collagen type-I precursor (Millipore), -PPARδ (Aviva System Biology; USA), -caspase-3, -AMPK or -APMK-P (Cell Signalling)]. Anti-GAPDH (Sigma) was used as loading control. Then, secondary antibodies (GE Healthcare) were used for chemo-luminescence development. A representative gel of at least three independent experiments with the semi-quantification score (n-fold) is shown in the figures. For quantification of soluble fibronectin, cultured cells were starved in 1% FBS (to prevent proteases activity) before stimulation, after which, cell media were collected, centrifuged (12,000 rpm at 4°C) to remove cell debris, and loaded (20 μg) on polyacrylamide gels. Since cells do not secrete GAPDH, Ponceau staining was used as a loading control. 

### Immunofluorescence (IF)

Fibronectin was localized in HL-1. After stimulation, cells were washed and fixed with 4% p-formaldehyde. Anti-fibronectin (Millipore) was added overnight at 4°C and developed with anti-rabbit FITC-linked goat antibody (Sigma). 4′,6-diamidino-2-phenylindole (DAPI, Sigma) was used for nuclear labeling. Fibronectin staining was quantified by Metamorph on ten randomly fields in at least three independent experiments. Cardiomyocyte size was quantified as previously described [24] as surface area from ten randomly chosen fields of actin-stained cells [with an anti-F-actin phalloidin antibody (Sigma)], in at least three independent experiments. Representative photographs with a bar scale were taken at 40x magnification under confocal microscopy. 

### Quantitative-PCR (QPCR)

Total RNA was extracted from homogenized ventricle (50 mg) or cultured cardiomyocytes by dissolving in Trizol reagent (Invitrogen). Equal amounts of RNA were reverse-transcripted to obtain the cDNA for multiplex QPCR. Mixture of QPCR was prepared as it follows: 33 ng of cDNA or 1:100 dilution of pre-amplified cDNA, 0.25 μl of gene expression assays [0.125 μl TGFß1 (Rn00572010_m1), CTGF (Rn00573960_m1), BNP (Rn00580641_m1) or α-SMA (Rn00570060_g1) Fam-fluorophore + 0.125 μl housekeeping gene eukaryotic ribosomic 18s vic-fluorophore (4310893E)], 5 μl premix buffer (polymerase and salts) and RNAase free water (Applied Biosystems). Amplification conditions were: 2’ at 50°C, 10’’ at 95°C and 40 cycles of 15’’ at 95°C and 1’ at 60°C. For GLP-1R detection, cDNA (100 ng) was pre-amplified (10’ at 95°C and 10 cycles of 15’’ at 95°C and 4’ at 60°C) with TaqMan PreAmp Master Mix (Applied Biosystems) before singleplex QPCR. All samples were prepared in triplicate to obtain their threshold cycle (Ct). If deviation for each triplicate were higher than 0.3 cycles, Ct was not considered. The relative expression for each gene was achieved following the model R=2^-ΔΔCt^. We show the quantification (-fold gene vs. 18s) of at least two QPCRs of all rats or three independent cultured cardiomyocytes experiments. 

### Statistical analysis

Data are expressed as mean±standard deviation. Multiple comparisons were performed by non-parametric Kruskal-Wallis test followed by a Mann-Whitney test. A two-tailed p<0.05 was considered significant.

## Results

### Characterization of the GK associated T2DM model

**Figure 1:**
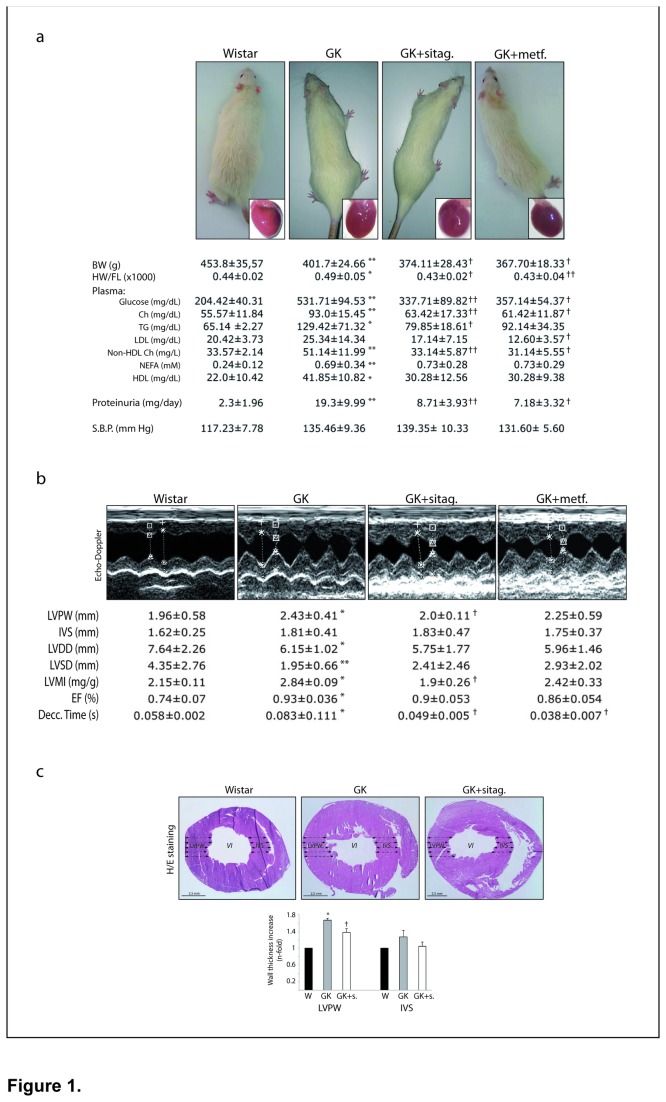
Sitagliptin and metformin reduced HW/FL, hyperglycemia, hyperlipidemia and proteinuria in GK rats. (**a**) Physical and plasmatic parameters were evaluated in the rats (n=10, each group). Representative photographs of rats and hearts for each group are also shown. BW, body weight; HW, heart weight, FL, femur length; Ch, cholesterol, TG, triglycerides; LDL and HDL, low- and high-density lipoproteins; NEFA, non-esterified fatty acid; S.B.P., systolic blood pressure. (**b**) **Sitagliptin mitigated cardiac hypertrophy in GK rats**. The LV posterior wall (LVPW) and inter-ventricular septum (IVS) thicknesses, LV diastolic (LVDD) and LV systolic (LVSD) diameters, LV mass index (LVMI), ejection fraction (EF) and the deceleration time were achieved in the rats myocardia (n=10, each g roup). Representative Echo-Doppler images for each group of rats are also shown (top). (**c**) Hematoxilin/Eosin staining of rat hearts with the corresponding semi-quantitative score of LVPW and IVS thicknesses. *p<0.05 and **p<0.01 vs. wistar. †p<0.05 and ††p<0.01 vs. GK rats.

 After 10 weeks of treatments, the patho/physiological parameters of the experimental model are shown in Figure 1a. GK rats showed elevated circulating levels of glucose, lipid profile [cholesterol (Ch), triglycerides (TG), non-HDL Ch, non-esterified fatty acids (NEFA) and high-density lipoproteins (HDL)], and proteinuria, compared to control hearts. GK also exhibited a modest but significant increase in heart-to-femur length ratio (HW/FL). Interestingly, both sitagliptin and metformin attenuated hyperglycemia, hyperlipidemia and proteinuria, and restored HW/FL (Figure 1a). Plasma ions (Na^+^, Cl^-^ and K^+^), markers of severe renal (urea, blood urea nitrogen, creatin and albumin) and liver (ASAT and ALAT) injury (not shown), and systolic blood pressure remained within the normal ranges in all groups of rats. 

### Sitagliptin attenuated the ventricular thickness, cell hypertrophy and cell-death of GK hearts

By Echo-Doppler (Figure 1b), GK showed a significant increase of LV posterior wall (LVPW) thickness and LV mass index (LVMI), resulting in a decreased LV diastolic (LVDD) and systolic (LVSD) diameters. Deceleration time was also prolonged in GK, suggesting diastolic dysfunction, and the ejection fraction (EF) was elevated. Sitagliptin treatment significantly reduced the LVPW, LVMI and deceleration time (Figure 1b). Metformin exhibited a decreased deceleration time and a non-significant trend to restore LVPW and LVMI. Cardiac wall thickness was also analysed in sitagliptin-treated hearts stained with H/E (Figure 1c). We confirmed the increase of LVPW in the GK myocardium and its diminution by sitagliptin. Moreover, we measured the cardiomyocyte diameters in this LVPW area. GK showed a significant increase of the cross-sectional area (1.41±0.04-fold vs. 1.0±0.03 wistar, p<0.05), which was reduced after sitagliptin (1.14±0.09-fold vs. wistar, p<0.05). 

**Figure 2:**
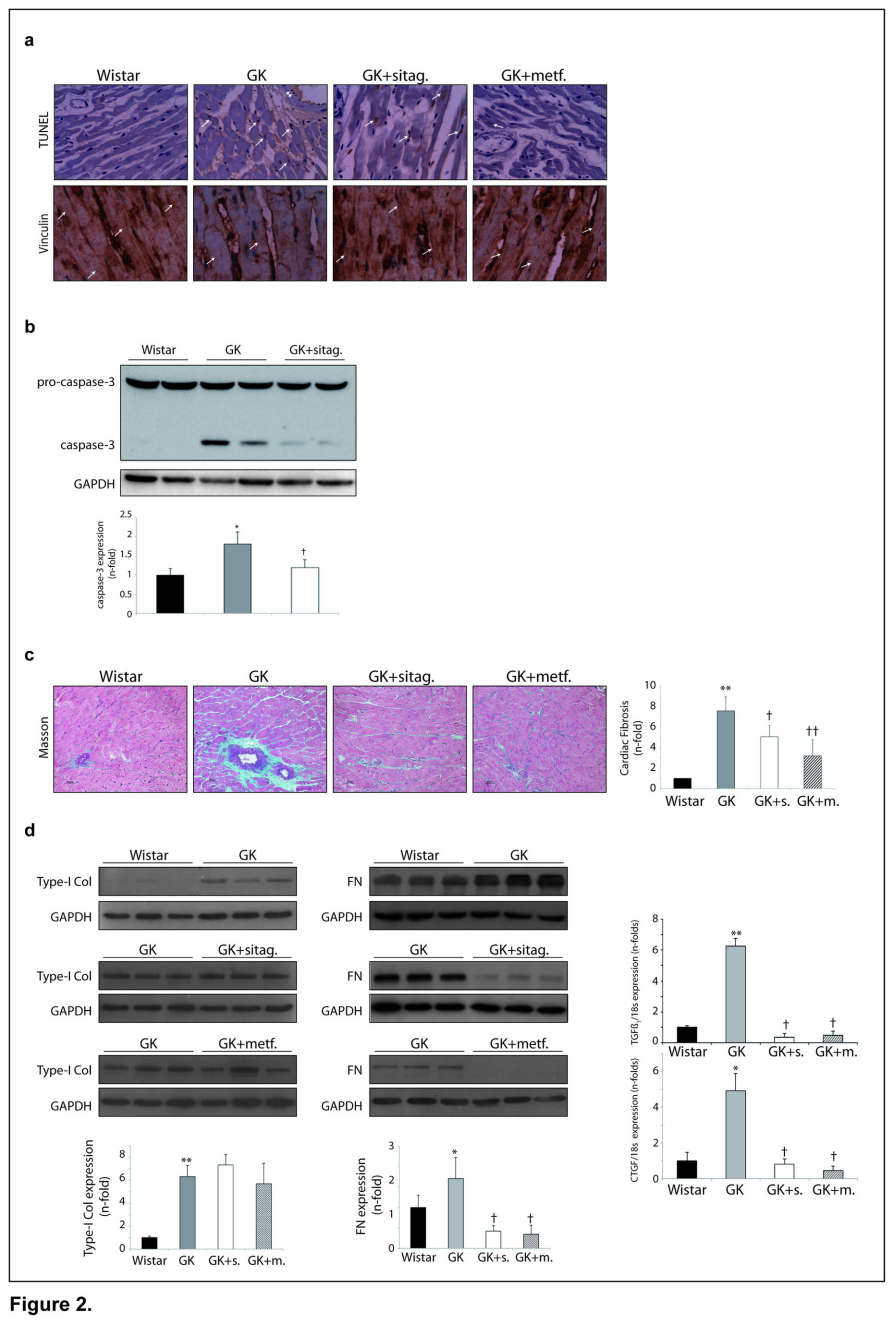
Sitagliptin and metformin reduced T2DM-associated cell-death and fibrosis in the heart. (**a**) By TUNEL, detection of apoptotic cells in the myocardium (see arrows) and heart vessel (see arrowheads). At the bottom, a typical striated-like pattern immunostaining of vinculin (see arrows). (**b**) Caspase-3 expression in the hearts. (**c**) Masson staining for wistar, GK and GK-treated hearts showing ECM accumulation (green-blue staining) (n=10, each group). (**d**) ECM protein [pro-type-I collagen and fibronectin (FN)] levels, and pro-fibrotic mRNA expression (TGFß_1_ and CTGF) (n=10, each group). *p<0.05 and **p<0.01 vs. wistar. †p<0.05 and ††p<0.01 vs. GK rats.

In addition, we also observed an increase of death cells in the GK myocardium (142.5±5.5%-fold vs. wistar, p<0.05). GK exhibited apoptotic myocytes and fibroblasts, and also vascular cells (mainly endothelial cells) (Figure 2a). In this regard, GK up-regulated pro-apoptotic caspase-3 (Figure 2b). Furthermore, necrotic cells were identified by a loss of the cytoskeleton protein vinculin. In this sense, GK hearts showed a decrease of vinculin (Figure 2a). Interestingly, sitagliptin and metformin reduced both apoptotic (125.5±3.0%-fold and 98.3±5.2%-fold vs. GK, respectively. p<0.05) and necrotic cells in the GK myocardia (Figure 2b), which was confirmed by restoration of caspase-3 and vinculin (Figure 2a).

### Sitagliptin reduced myocardial fibrosis and pro-fibrotic factors

 Left ventricular myocardium in the wistar rats showed a normal architecture with regular interstitial space (Figure 2c). In contrast, abnormal myocardial architecture (cardiomyocyte disarray and increased interstitial space) was observed in the GK group. Masson trichrome staining detected a deposition of ECM within the interstitial and mainly peri-vascular areas (green-blue staining, Figure 2c). Interestingly, in both sitagliptin and metformin-treated rats, ECM accumulation was markedly reduced. Thus, we next focused on the fibrotic component by examining the expression of key cardiac ECM proteins. Pro-type-I collagen and fibronectin were found elevated in GK rats (Figure 2d, left). However, only fibronectin was reverted with both sitagliptin and metformin. Furthermore, the mRNA expression of pro-fibrotic inducers, such as transforming growth factor-ß_1_ (TGFß_1_) and its cofactor, connective tissue growth factor (CTGF), were also up-regulated in the GK myocardium and restored with both treatments (Figure 2d, right). 

### Sitagliptin increased GLP-1 secretion in fasting and glucose-overload states in GK rats

**Figure 3:**
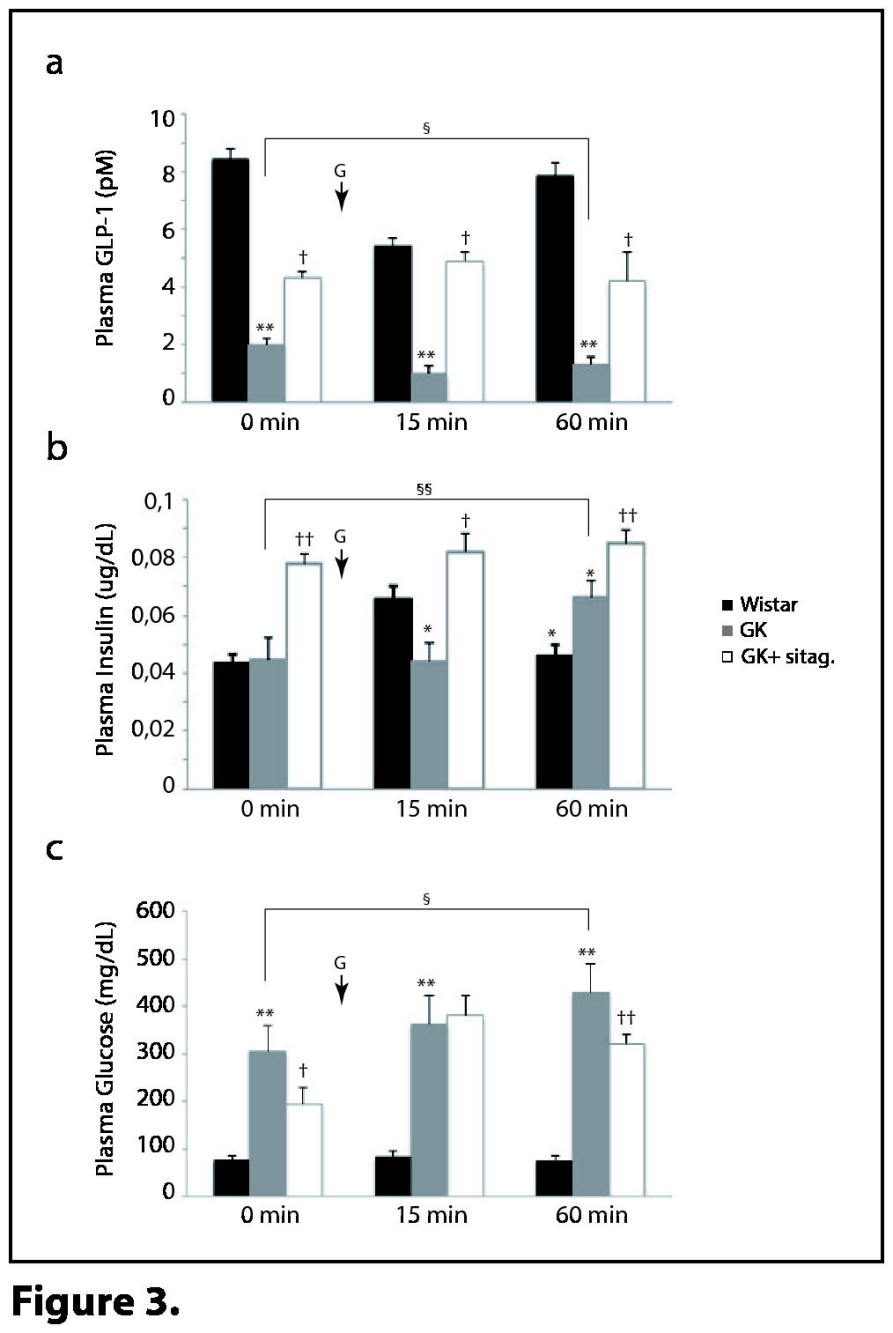
Sitagliptin improved glucose intolerance in GK **rats**. Plasma (**a**) GLP-1, (**b**) insulin and (**c**) glucose were evaluated in the rats before (fasting) and 15-min/60-min after glucose loading (n=10, each group). The G-black arrow indicates glucose-overload. *p<0.05 and **p<0.01 vs. wistar. †p<0.05 and ††p<0.01 vs. GK rats. §p<0.05 and §§p<0.01 vs. fasting state.

 Previous data had indicated that sitagliptin could increase plasma GLP-1 in a glucose-dependent manner [24,25]. By a glucose tolerance test, we observed in the fasting state (0 min) that GLP-1 levels were diminished in GK rats and moderately reverted after sitagliptin (Figure 3a), which may correlate with the increase of insulin (Figure 3b) and ameliorated glucose (Figure 3c) in these rats. Then, mainly after 60 min of glucose loading, GK showed decreased levels of GLP-1 (Figure 3a) and a delayed insulin response (Figure 3b), which could be responsible for the increasing concentration of plasma glucose (Figure 3c). However, also after 60 min, sitagliptin-treated rats kept higher levels of GLP-1 (Figure 3a) and insulin (Figure 3b), which may achieve for the slight but significant glycemic reduction (Figure 3c).

### Expression of cardiac GLP-1 receptors

**Figure 4:**
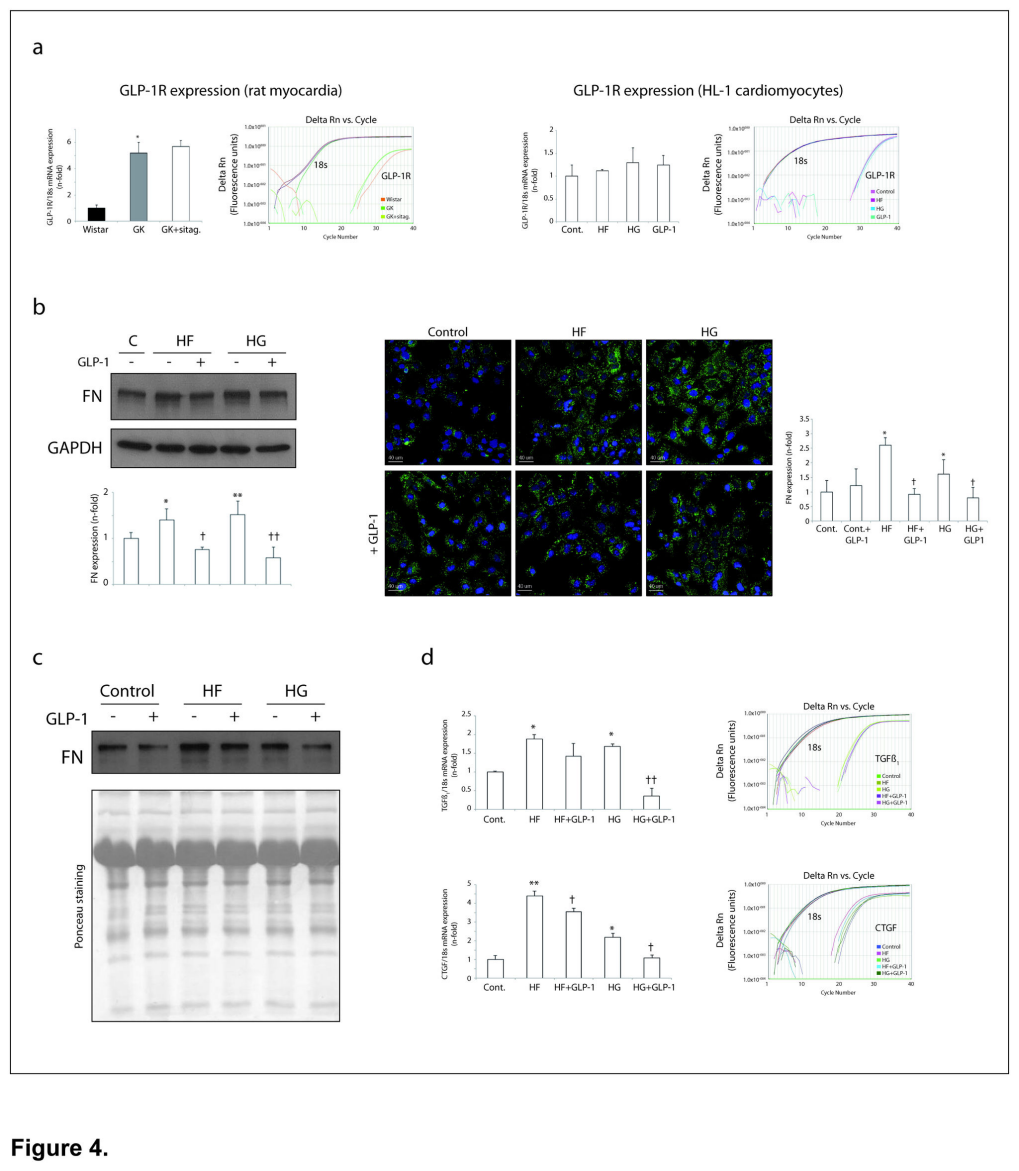
GLP-1 reduced pro-fibrotic molecules in HF- or HG-stimulated cardiomyocytes. (**a**) GLP1R expression in the GK model (left) (n=5, each group), and HL-1 stimulated cardiomyocytes (right). A representative QPCR-amplification plot of each rat or stimulated cell is also showed. (**b**) Intracellular (by WB and IF) and (**c**) secreted levels of fibronectin (FN) in GLP-1-pre-treated cardiomyocytes exposed to HF (0.25 mM) or HG (33 mM). (**d**) Pro-fibrotic expression (TGFß_1_ and CTGF) in stimulated cardiomyocytes. *p<0.05 and **p<0.01 vs. control. †p<0.05 and ††p<0.01 vs. HF or HG.

The existence of GLP-1 receptor (GLP-1R) has been described in the heart, specifically in cardiomyocytes but not in fibroblasts [8]. We indeed detected GLP-1R mRNA expression in the rat hearts (Figure 4a, left). GK exhibited higher GLP-1R expression than wistar, and sitagliptin did not modify this value. As expected, in contrast to cardiac fibroblast and TFB (not shown), we detected GLP-1R mRNA expression in HL-1 cardiomyocytes, and this was not significantly changed after 6h of HF, HG or GLP-1 incubation (Figure 4a, right). The presence of GLP-1R in the heart and cardiomyocytes suggested a direct role of GLP-1 in the DCM.

### GLP-1 regulated HF- and/or HG-induced pro-fibrosis, hypertrophy and cell-death in cardiomyocytes

An excess of FFA and glucose are main components of the hyperlipidemic and hyperglycemic milieu in DCM [2,22]. We stimulated HL-1 cardiomyocytes with both compounds and focused on the pro-fibrotic response. After 24h, high-concentration of FFA (HF) or glucose (HG) triggered fibronectin expression (Figure 4b, by WB and IF) and secretion to the cultured media (Figure 4c). When both HF and HG were added together, fibronectin was not significantly further stimulated (not shown). In concordance, the mRNA expression of pro-fibrotic cytokines, such as TGFß_1_ and CTGF, were also up-regulated after 6h of HF or HG (Figure 4d). Then, since sitagliptin increased GLP-1 plasma levels (Figure 3a) and reduced myocardial fibrosis in GK rats (Figure 2a), we assayed whether GLP-1 may modulate the pro-fibrotic response to HF and HG in cardiomyocytes. GLP-1 pre-treatment (1 nM) attenuated fibronectin expression (Figure 4b, by WB and IF) and secretion (Figure 4c) in HF or HG-induced cells. GLP-1 alone did not significantly alter fibronectin content in control cells (Figure 4b-c). Moreover, GLP-1 decreased pro-fibrotic TGFß_1_ transcripts after HG, and CTGF after HF or HG incubation (Figure 4d). 

In addition, we studied whether HF and HG could induce pro-hypertrophic and lethal influences on cardiomyocytes and whether GLP-1 may alleviate these effects. After 24h, HF increased the cardiomyocyte size (167.4± 13.1% vs. control; Figure S1a, left), and the mRNA expression of brain natriuretic peptide (BNP) (Figure S1a, right). HG also triggered cardiomyocyte hypertrophy (183.0±9.4% vs. control; Figure S1a, left), and the mRNA levels of BNP and cytoskeleton smooth muscle α-actin (α-SMA) (Figure S1a, right). By other hand, only HF induced cell death in cardiomyocytes (145.2±7.8% vs. control. Figure S1b, left) and cardiac fibroblasts (136.9±4.3% vs. control. Figure S1b, right). Moreover, HF increased caspase-3 expression and the release of glucose 6-phosphate dehydrogenase (G6PD), a marker of necrosis (Figure S1c). Interestingly, GLP-1 pre-treatment mitigated HF-/HG-induced hypertrophy (97.2±7.5% and 94.3±6.9% vs. HF and HG, respectively) and HF-induced cardiomyocyte and cardiac fibroblast death (123.6±8.3% and 115.2±6.3% vs. control), and decreased the related pro-hypertrophic and apoptotic/necrotic markers (Figure S1a, c). 

### Insulinotropic inactive GLP-1(9-36) exhibited similar cardioprotective effects than GLP-1

**Figure 5:**
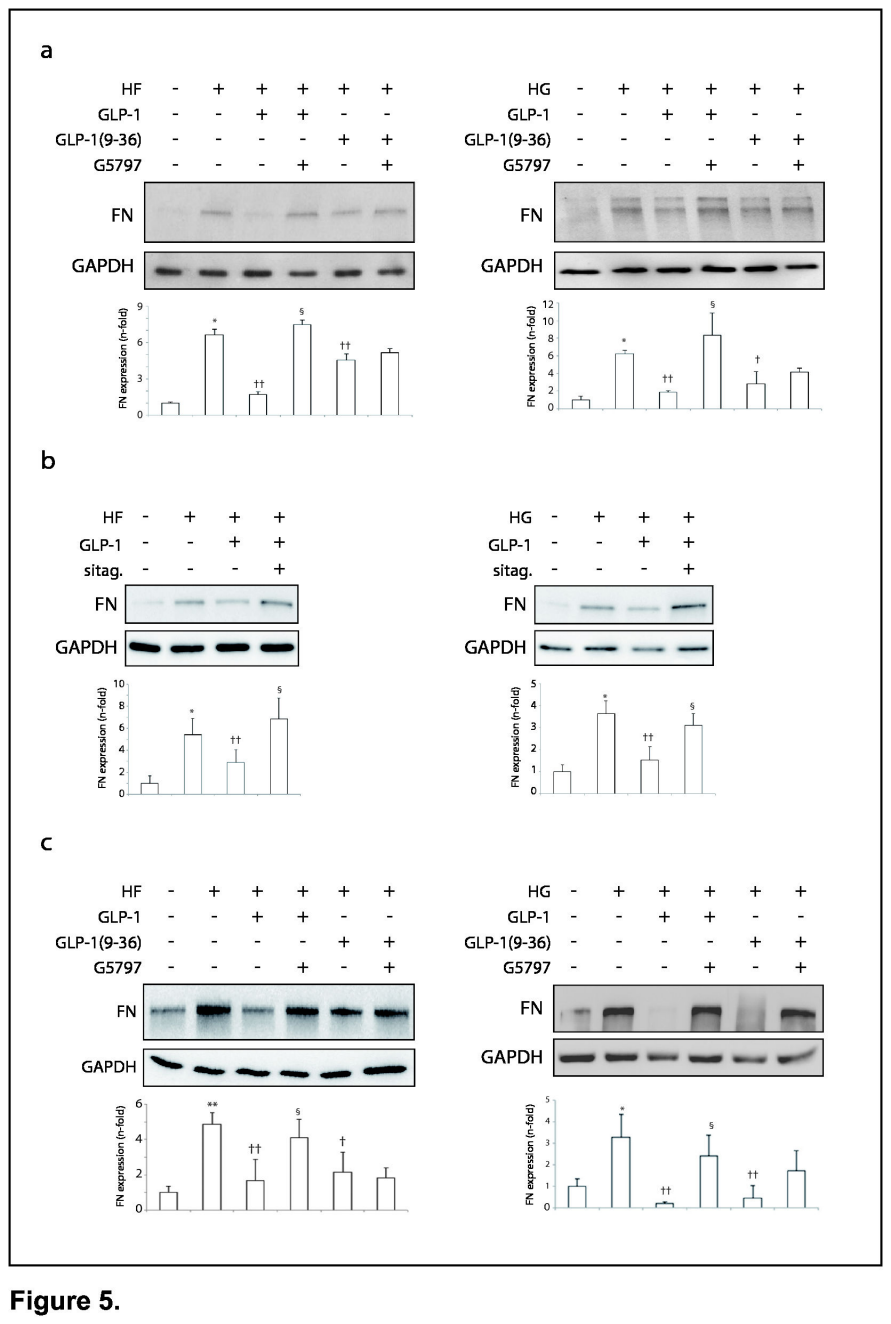
Anti-fibrotic effects of GLP-1 and GLP-1(9-36) on HF- or HG-incubated cardiomyocytes and cardiac fibroblasts. Implication of PPARδ. (a) Fibronectin levels in GLP-1 and GLP-1(9-36) pre-treated cardiomyocytes exposed to HF (left) or HG (right). Some cells were also incubated with a PPARδ antagonist (G5797). (**b**) Pre-treatment of sitagliptin in GLP-1 +/- HF-/HG-stimulated cardiomyocytes. (**c**) Fibronectin expression was also examined in cardiac fibroblast. *p<0.05 and **p<0.01 vs. control. †p<0.05 and ††p<0.01 vs. HF or HG. §p<0.05 vs. HF+GLP-1 or HG+GLP-1.

GLP-1 cannot be produced by cardiac cells [26, 27]. However, in cultured cardiomyocytes, exogenous GLP-1 might be converted to GLP-1(9-36) by the DPP-IV activity [9, 27]. Then, we assayed whether the anti-apoptotic/necrotic, -hypertrophic and -fibrotic effects of GLP-1 may be a consequence of GLP-1(9-36) actions. In a similar manner to GLP-1, GLP-1(9-36) reduced the expression of caspase-3, G6PD (Figure S1c), BNP/α-SMA (Figure S1a) and fibronectin (Figure 5a, 3^rd^ and 5^th^ lanes) in HF- and/or HG-stimulated cardiomyocytes. Moreover, the anti-fibrotic effect of GLP-1 was reversed by sitagliptin pre-treatment, suggesting a direct role of GLP-1(9-36) (Figure 5b, 4^th^ lanes). As expected, sitagliptin alone did not affect the pro-fibrotic proprieties of HL-1 after both stimuli (not shown). 

In addition, although fibroblasts did not express GLP-1R (our data and [8]), we tested whether they could respond to GLP-1 isoforms by different receptors, as previously suggested [27]. HF and HG also triggered fibronectin expression in cardiac fibroblasts, and both GLP-1 and GLP-1(9-36) reduced also these levels (Figure 5c, 3^rd^ and 5^th^ lanes). A similar result was seen in TFB cells and H9c2 cardiomyocytes, which also lack GLP-1R (not shown).

### PPARδ mediated the anti-fibrotic actions of GLP-1 stimulation

**Figure 6:**
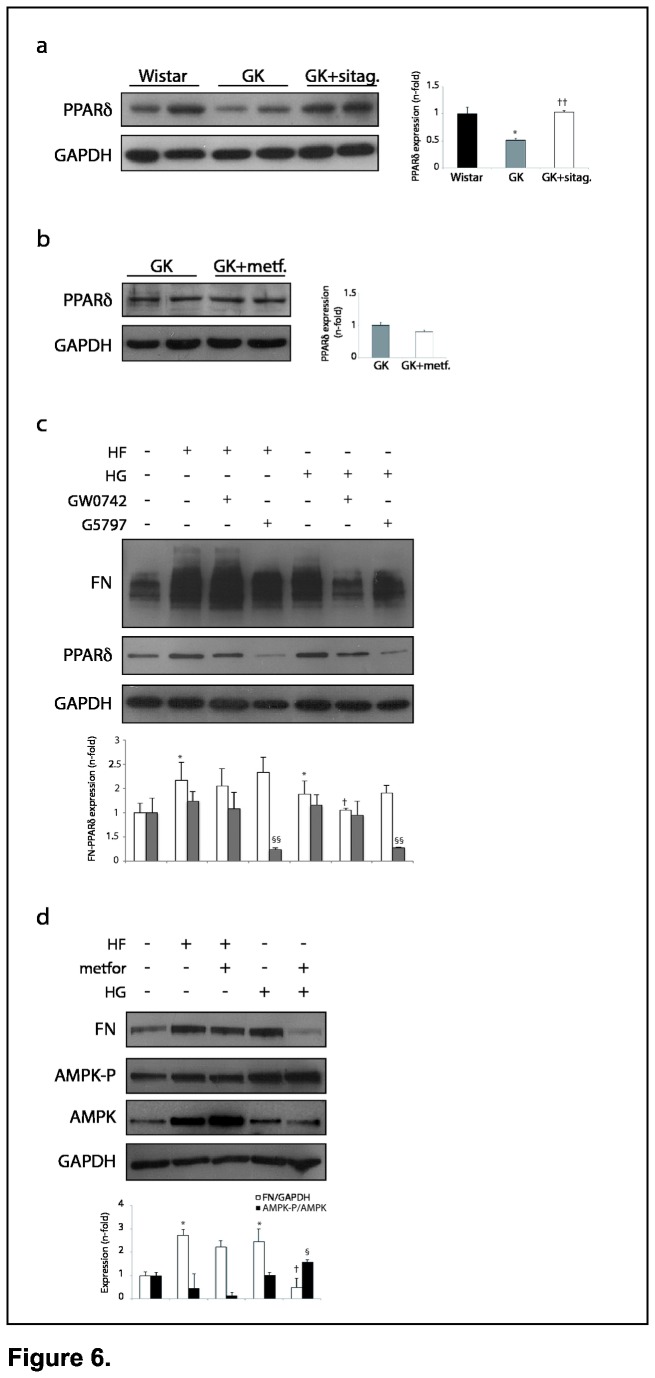
PPARδ and fibronectin expression in the heart. Representative blots of PPARδ levels in (**a**) wistar, GK, GK-sitagliptin and (**b**) GK-metformin rats (n=10, each group). (**c**) Fibronectin and PPARδ expression in HF-/HG-incubated cardiomyocytes pre-treated with a PPARδ agonist (GW0742) or antagonist (G5797). *p<0.05 vs. control. †p<0.05 and ††p<0.01 vs. GK or HG. §§p<0.01 vs. HF or HG. (**d**) **Reduction of fibronectin in metformin-treated fibroblasts**. Fibronectin, AMPK-phosphorylated and AMPK levels in HF-/HG-incubated TFB +/- metformin. *p<0.05 vs. control. †p<0.05 vs. HF or HG. §p<0.05 vs. HG.

A proposed GLP-1 downstream mediator could be the peroxisome proliferator activating receptor-δ (PPARδ) [28, 29]. In this regard, a significant decrease of PPARδ levels was noted in the GK myocardium, and this effect was normalized by sitagliptin (Figure 6a), but not metformin (Figure 6b). Since PPARδ had demonstrated anti-fibrotic proprieties in the heart [12, 13], but its role on the diabetic scenario was unknown, we first tested whether PPARδ activation may affect fibronectin up-regulation induced by HF or HG. Intriguingly, a PPARδ agonist (GW0742) pre-treatment attenuated fibronectin levels only in HG-stimulated cardiomyocytes (Figure 6c, 6^th^ lane). Thus, in concordance, the anti-fibrotic effect of GLP-1, but not GLP-1(9-36), was significantly reverted by a specific PPARδ antagonist (G5797) in HF- or HG-stimulated cardiomyocytes (Figure 5a, 4^th^ lanes). Similar data were also observed in cardiac fibroblasts (Figure 5c, 4^th^ lanes). 

### Direct protective effects of metformin on cardiac cells

Since both sitagliptin and metformin similarly reduced fibrosis in the GK heart, and GLP-1 triggered anti-fibrotic actions on cultured cardiomyocytes and cardiac fibroblasts, we tested whether metformin may also induce direct anti-fibrotic influence on cardiac cells. Indeed, we observed a reduction of fibronectin up-regulation in HG-stimulated cardiomyocytes (not shown) and TFB fibroblasts (Figure 6d) pre-treated with metformin. In addition, a known metformin-mediator playing key roles in DCM [30], AMP-activated protein kinase (AMPK), was also activated (phosphorylated) in HG+metformin cells. However, metformin did not diminish fibronectin levels after HF, nor increased phosphorylated-AMPK despite AMPK was over-expressed (Figure 6d). In this regard, AMPK could be used for deacetylation processes in mitochondria biogenesis [30]. Of note, metformin also reduced the expression of caspase-3, and the release of G6PD, but not BNP/α-SMA mRNA overexpression, in HF- and/or HG-stimulated cells (Figure S1 a, c).

## Discussion

T2DM *per se* can damage the heart. Non-hypertensive non-obese GK rats exhibited an accumulation of ECM in the myocardium, and up-regulation of pro-fibrotic TGFß_1_, CTGF and main ECM components such as type-I collagen and fibronectin. These data are in consonance with previous results in type-I diabetic rodents [31, 32]. Importantly, cardiac fibrosis may be induced by direct stimulation of released cytokines and/or indirectly by a mechanism of replacing died cells, thus preserving the structural integrity of the myocardium. However, fibrosis may increase hypertrophy and cardiac dysfunction [2, 4, 33]. In this regard, GK showed cardiac and myocellular hypertrophy that along with data suggest diastolic dysfunction. Unexpectedly, EF was elevated in GK rats. This could be a consequence of the reduction of cavity volume and near-complete emptying of the ventricle in order to maintain cardiac output, as occurs in hypertrophied hearts [34]. In this sense, human and experimental diabetic-associated heart failure has been also described without a reduction of EF [35, 36]. 

**Figure 7:**
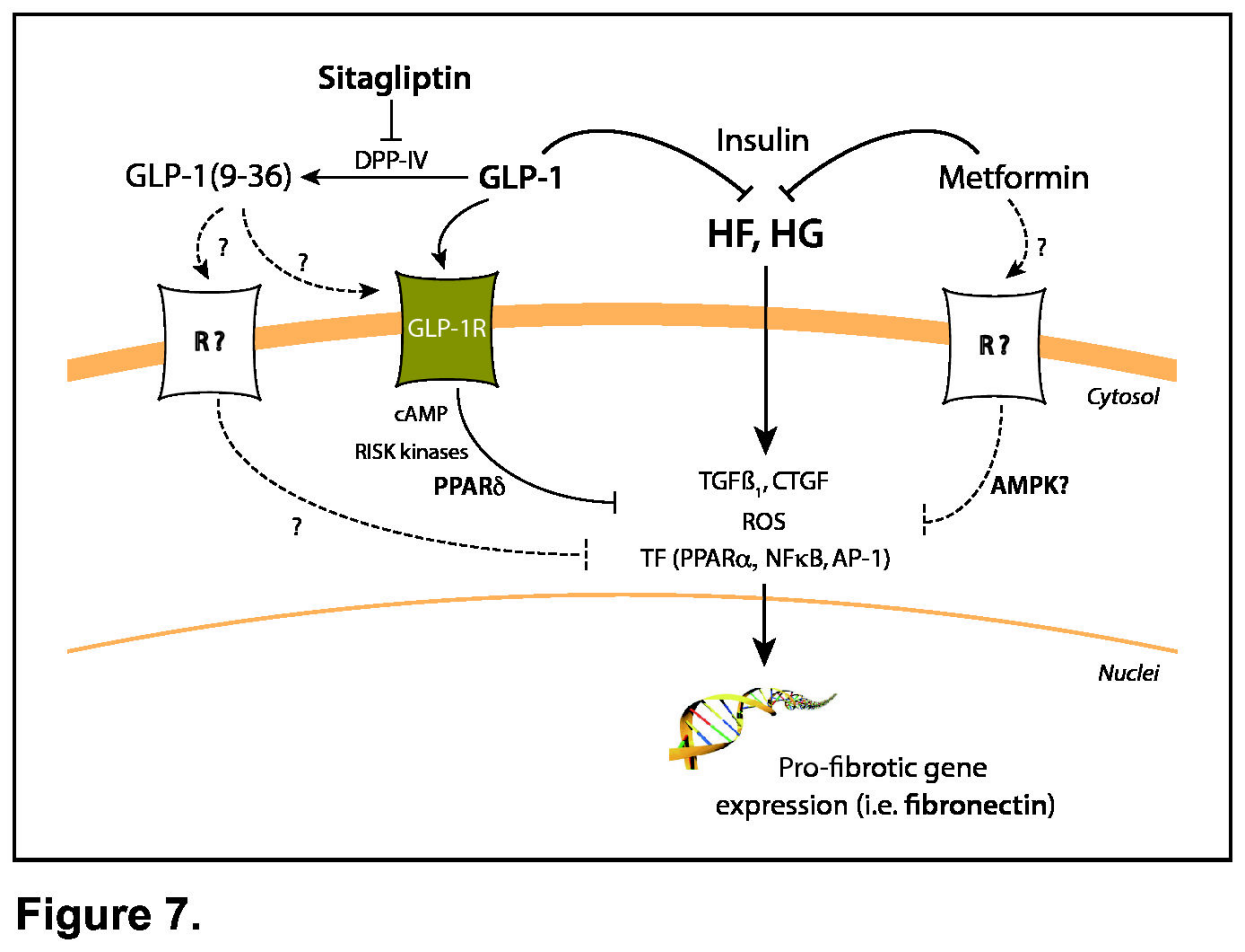
Hypothesised anti-fibrotic mechanisms activated in sitagliptin- or metformin-treated GK hearts. In T2DM cardiac cells, two major components such as HF and HG can trigger pro-fibrotic molecules (i.e. fibronectin) through activation of specific mediators [TGFß_1_, CTGF, ROS and transcription factors (TF)]. This response may be primarily attenuated by the GLP-1- or metformin-induced insulinotropic effect. However, a direct anti-fibrotic action of GLP-1GLP-1RPPARδ or metforminAMPK may also occur. In addition, the GLP-1 truncated isoform [GLP-1(9-36)] may also conserve anti-fibrotic properties by a GLP-1R-dependent or independent way. R; unknown receptor.

To date, there is not a specific treatment for DCM-associated fibrosis. DPP-IV inhibitors, as sitagliptin, suppress the DPP-IV activity and consequently, prolong GLP-1 half-life. GLP-1 accounts for at least 50-70% of postprandial insulin secretion, improving the glycemic control by a glucose-dependent mechanism [5]. In our data, sitagliptin reduced hyperglycemia and glucose intolerance. Moreover, we described a lipid-lowering influence. Other DPP-IV inhibitors have also showed hypolipidemic effects on T2DM patients [37, 38]. The mechanisms for this lipidemic control may be related to the GLP-1 effect on lipid absorption [39], metabolism [40] and/or PPARs activation (see later). More interestingly, sitagliptin attenuated cardiac apoptosis/necrosis, hypertrophy and fibrosis in experimental T2DM. These effects may respond to an increased insulin response by plasma GLP-1 stabilization. In fact, in these rats metformin induced similar anti-apoptotic/necrotic, -hypertrophic and -fibrotic actions, and improved cardiac function. However, other sitagliptin-associated cardioprotective actions have been reported in non-diabetic injuries. Sitagliptin reduced the infarct size in mice with ischemia-reperfusion [41], and diminished post-ischemic stunning in patients with coronary artery disease and preserved LV function [42]. Also, a GLP-1-analogue treatment improved cardiac function in non-diabetic infarcted patients. Thus, besides its insulin-dependent glycemic and lipidemic control, sitagliptin might play salutary roles by direct actions of GLP-1 on cardiac cells [6]. In this regard, although GLP-1 is expressed in brain, pancreas or intestine, but not heart [26], the presence of GLP-1R have been demonstrated in cardiomyocytes [8], and GLP-1R knockout mice displayed impaired LV contractibility and diastolic function [45]. We have confirmed GLP-1R expression in rat hearts and HL-1 cardiomyocytes, and also, we have detected alleviation of the pro-apoptotic/necrotic, hypertrophic and fibrotic expression in GLP-1 pre-treated cardiomyocytes exposed to HF and/or HG. Previous data indicated also anti-apoptotic actions of GLP-1 in HL-1 cells [18]. However, these effects may respond also to the action of its metabolite GLP-1(9-36). In fact, we found similar effects on HF- and/or HG-stimulated cardiac cells for GLP-1(9-36), and sitagliptin reversed at least the anti-fibrotic action of GLP-1. GLP-1(9-36) may also promote cardioprotection in T2DM. In this regard, GLP-1(9-36) exerted anti-oxidant effects on cardiac and vascular cells [28, 46]. Thus, since metformin and GLP-1(9-36) induce similar anti-apoptotic/necrotic, -hypertrophic and -fibrotic actions than GLP-1, the cardioproptective effects observed after sitagliptin administration should be firstly explained by its insulinotropic proprieties. Moreover, we have also described direct anti-apoptotic/necrotic and anti-fibrotic effects of metformin on HF- or HG-stimulated cardiac cells, likely involving AMPK activation. Similar results were found in H_2_O_2_- and TGFß-incubated cardiomyocytes and fibroblasts, respectively [47] [48]. However, these *in vitro* approaches may not accurately reproduce *in vivo* GLP-1 secretion and DPP-IV inhibition (or metformin cardiac bioavailability), and the large population of cardiac non-myocytes/fibroblasts that express pro-fibrotic factors could differentially respond to GLP-1 isoforms (or metformin). Further investigations studying the potential interactions between GLP-1R and metformin-linked mediators (i.e. AMPK) could be of high interest (Figure 7). In this sense, GLP-1 may also directly counteract the pro-oxidative, inflammatory and apoptotic activities induced by angiotensin-II [43,44].

Moreover, the cellular mechanisms activated by GLP-1 isoforms are not elucidated. GLP-1 and GLP-1(9-36) can signal by GLP-1R or different receptors [28, 46]. Indeed, we observed that although cardiac fibroblasts did not express GLP-1R, both GLP-1 and GLP-1(9-36) reduced the pro-fibrotic expression after HF or HG. Downstream, cardiac cAMP-dependent RISK kinases have been involve in the GLP-1R signalling to activate different transcription factors, such as PPARδ [49-52]. PPARδ, a highly expressed nuclear receptor in cardiac cells, promotes healthy activities in the diabetic heart by up-regulation of FFA-oxidation enzymes [53]. Additionally, PPARδmay also control the pro-fibrotic expression [54]. We observed a lessening of HG-induced fibronectin expression after PPARδ-agonist administration. In HF-stimulated cells a PPARδ-agonist did not change fibronectin levels possibly because PPARδ can be also a mediator of FFA signaling. Wagner et al. described a reduction of myocardial collagen in PPARδ-agonist treated mice [52]. In addition, we noted that sitagliptin, but not metformin, returned PPARδ levels in GK hearts. Also, in HF-/HG-stimulated cardiomyocytes, GLP-1, but not GLP-1(9-36), reduced pro-fibrotic factors in a PPARδ-dependent way. In this regard, a GLP-1-analogue increased myocardial PPARδ and reduced apoptosis in infarcted mice [29]. Altogether, at least for the anti-fibrotic effect, GLP-1 could show more affinity for GLP-1R than GLP-1(9-36), and this receptor may be linked to PPARδ activation (Figure 7). However, further experiments are required to establish the precise assembly of this phenomenon. 

### Study limitations

Another incretin, termed glucose-dependent insulinotropic polypeptide (GIP), will be increased after DPP-IV inhibition. However, GIP is much less active and its receptor is profoundly decreased under hyperglycemia [55]. Also, we cannot exclude that other potential DPP-IV targets, such as stromal cell-derived factor-1 chemokine [5] [28], might affect some cardiovascular responses. 

## Conclusions

In chronic experimental DCM, the apoptotic and fibrotic responses may promote myocardial hypertrophy and remodelling. However, DPP-IV inhibitors as sitagliptin, through plasma GLP-1 stabilization and insulin control of hyperglycemia/lipidemia, reduced the cardiac pro-apoptotic/necrotic, hypertrophic and fibrotic expression in a similar way to metformin. However, in the presence of high concentrations of palmitate or glucose, cultured cardiac cells demonstrated a direct effect of GLP-1PPARδ, GLP-1(9-36) and metformin on related factors through GLP-1R or distinct receptors. Furthermore, the cardioprotective actions of GLP-1(9-36) suggests additional benefits of GLP-1 analogues, which do not interfere with the physiological GLP-1 degradation, over the ones from DPP-IV inhibitors in diabetic and, interestingly, non-diabetic cardiomyopathies.

## Supporting Information

Figure S1 **GLP-1 isoforms alleviated the pro-hypertrophic and lethal responses after HF- and/or HG-stimulation in cardiac cells.** (a, **left**) HF-/HG-incubated cardiomyocytes were pre-treated with GLP-1, and F-actin (red) was detected by IF for cell-size quantification. (a, **right**) Some cells were used for BNP and α-SMA mRNA detection. (**b**) Dying cardiomyocytes (left) or cardiac fibroblasts (right) were observed under microscopy after HF/HG-stimulation and GLP-1 pre-treatment. (**c**) Caspase-3 expression and the release of G6PD to the cultured media was measured in stimulated cardiomyocytes. *p<0.05 and **p<0.01 vs. control. †p<0.05 and ††p<0.01 vs. HF or HG.
(AI) Click here for additional data file.

## References

[1] American Diabetes Association (2009) Standards of medical care in diabetes. Diabetes Care, 32 Suppl 1: S13–S61. doi:10.2337/dc09-S013. PubMed: 19118286. 19118286 PMC2613589

[2] Aneja A, Tang WHW, Bansilal S, Garcia MJ, Farkouh ME (2008) Diabetic cardiomyopathy: insights into pathogenesis, diagnostic challenges, and therapeutic options. Am J Med, 121: 748–757. doi:10.1016/j.amjmed.2008.03.046. PubMed: 18724960. 18724960

[3] Chen S, Evans T, Mukherjee K, Karmazyn M, Chakrabarti S (2000) Diabetes-induced myocardial structural changes: role of endothelin-1 and its receptors. J Mol Cell Cardiol, 32: 1621–1629. doi:10.1006/jmcc.2000.1197. PubMed: 10966825. 10966825

[4] Mizushige K, Yao L, Noma T, Kiyomoto H, Yu Y et al. (2000) Alteration in left ventricular diastolic filling and accumulation of myocardial collagen at insulin-resistant prediabetic stage of a type II diabetic rat model. Circulation, 101: 899–907. doi:10.1161/01.CIR.101.8.899. PubMed: 10694530. 10694530

[5] Drucker DJ (2007) Dipeptidyl peptidase-4 inhibition and the treatment of type 2 diabetes: preclinical biology and mechanisms of action. Diabetes Care, 30: 1335–1343. doi:10.2337/dc07-0228. PubMed: 17337495. 17337495

[6] Nikolaidis LA, Mankad S, Sokos GG, Miske G, Shah A et al. (2004) Effects of glucagon-like peptide-1 in patients with acute myocardial infarction and left ventricular dysfunction after successful reperfusion. Circulation, 109: 962–965. doi:10.1161/01.CIR.0000120505.91348.58. PubMed: 14981009. 14981009

[7] Sokos GG, Nikolaidis LA, Mankad S, Elahi D, Shannon RP (2006) Glucagon-like peptide-1 infusion improves left ventricular ejection fraction and functional status in patients with chronic heart failure. J Card Fail, 12: 694–699. doi:10.1016/j.cardfail.2006.08.211. PubMed: 17174230. 17174230

[8] Ban K, Noyan-Ashraf MH, Hoefer J, Bolz S-S, Drucker DJ et al. (2008) Cardioprotective and vasodilatory actions of glucagon-like peptide 1 receptor are mediated through both glucagon-like peptide 1 receptor-dependent and -independent pathways. Circulation, 117: 2340–2350. doi:10.1161/CIRCULATIONAHA.107.739938. PubMed: 18427132. 18427132

[9] Mudaliar S, Henry RR (2012) The incretin hormones: from scientific discovery to practical therapeutics. Diabetologia, 55: 1865–1868. doi:10.1007/s00125-012-2561-x. PubMed: 22555471. 22555471

[10] Hui H, Tang YG, Zhu L, Khoury N, Hui Z et al. (2010) Glucagon like peptide-1-directed human embryonic stem cells differentiation into insulin-producing cells via hedgehog, cAMP, and PI3K pathways. Pancreas, 39: 315–322. doi:10.1097/MPA.0b013e3181bc30dd. PubMed: 19924023. 19924023

[11] Glauser DA, Brun T, Gauthier BR, Schlegel W (2007) Transcriptional response of pancreatic beta cells to metabolic stimulation: large scale identification of immediate-early and secondary response genes. BMC Mol Biol, 8: 54. doi:10.1186/1471-2199-8-54. PubMed: 17587450. 17587450 PMC1914353

[12] Wagner K-D, Wagner N (2010) Peroxisome proliferator-activated receptor beta/delta (PPARbeta/delta) acts as regulator of metabolism linked to multiple cellular functions. Pharmacol Ther, 125: 423–435. doi:10.1016/j.pharmthera.2009.12.001. PubMed: 20026355. 20026355

[13] Zhang H, Pi R, Li R, Wang P, Tang F et al. (2007) PPARbeta/delta activation inhibits angiotensin II-induced collagen type I expression in rat cardiac fibroblasts. Arch Biochem Biophys, 460: 25–32. doi:10.1016/j.abb.2007.01.028. PubMed: 17346664. 17346664

[14] Ostenson C-G, Efendic S (2007) Islet gene expression and function in type 2 diabetes; studies in the Goto-Kakizaki rat and humans. Diabetes Obes Metab, 9 Suppl 2: 180–186. doi:10.1111/j.1463-1326.2007.00787.x. PubMed: 17919192. 17919192

[15] Tahara A, Matsuyama-Yokono A, Nakano R, Someya Y, Hayakawa M et al. (2009) Antihyperglycemic effects of ASP8497 in streptozotocin-nicotinamide induced diabetic rats: comparison with other dipeptidyl peptidase-IV inhibitors. Pharmacol Rep, 61: 899–908. PubMed: 19904014. 19904014 10.1016/s1734-1140(09)70147-1

[16] Orskov C, Holst JJ (1987) Radio-immunoassays for glucagon-like peptides 1 and 2 (GLP-1 and GLP-2). Scand J Clin Lab Invest, 47: 165–174. doi:10.3109/00365518709168885. PubMed: 3576119. 3576119

[17] Escudero EM, Camilión de Hurtado MC, Pérez NG, Tufare AL (2004) Echocardiographic assessment of left ventricular midwall mechanics in spontaneously hypertensive rats. Eur J Echocardiogr, 5: 169–175. doi:10.1016/j.euje.2003.11.004. PubMed: 15147658. 15147658

[18] Ravassa S, Zudaire A, Carr RD, Díez J (2011) Antiapoptotic effects of GLP-1 in murine HL-1 cardiomyocytes. Am J Physiol Heart Circ Physiol, 300: H1361–H1372. doi:10.1152/ajpheart.00885.2010. PubMed: 21278133. 21278133

[19] Claycomb WC, Lanson NA, Stallworth BS, Egeland DB, Delcarpio JB et al. (1998) HL-1 cells: A cardiac muscle cell line that contracts and retains phenotypic characteristics of the adult cardiomyocyte. Proc Natl Acad Sci U S A, 95: 2979–2984. doi:10.1073/pnas.95.6.2979. PubMed: 9501201. 9501201 PMC19680

[20] Martín R, Miana M, Jurado-López R, Martínez-Martínez E, Gómez-Hurtado N et al. (2012) DIOL triterpenes block profibrotic effects of angiotensin II and protect from cardiac hypertrophy. PLOS ONE. 7(7): e41545 PubMed: 22844495. 22844495 10.1371/journal.pone.0041545 PMC3402387

[21] Strutz F, Okada H, Lo CW, Danoff T, Carone RL et al. ( 7 1995) Identification and characterization of a fibroblast marker: FSP1. J Cell Biol 7;130 (2): 393-405. doi:10.1083/jcb.130.2.393. PubMed: 7615639. 7615639 PMC2199940

[22] Hickson-Bick DLM, Sparagna GC, Buja LM, McMillin JB (2002) Palmitate-induced apoptosis in neonatal cardiomyocytes is not dependent on the generation of ROS. Am J Physiol Heart Circ Physiol, 282: H656–H664. PubMed: 11788415. 11788415 10.1152/ajpheart.00726.2001

[23] Ishibashi Y, Matsui T, Takeuchi M, Yamagishi S (2011) Sitagliptin augments protective effects of GLP-1 against advanced glycation end product receptor axis in endothelial cells. Horm Metab Res, 43: 731–734. doi:10.1055/s-0031-1284383. PubMed: 21932180. 21932180

[24] Ares-Carrasco S, Picatoste B, Camafeita E, Carrasco-Navarro S, Zubiri I et al. (2012) Proteome changes in the myocardium of experimental chronic diabetes and hypertension: Role of PPARα in the associated hypertrophy. J Proteomics, 75: 1816–1829. doi:10.1016/j.jprot.2011.12.023. PubMed: 22234359. 22234359

[25] Dhillon S (2010) Sitagliptin: a review of its use in the management of type 2 diabetes mellitus. Drugs, 70: 489–512. doi:10.2165/11203790-000000000-00000. PubMed: 20205490. 20205490

[26] Mojsov S, Heinrich G, Wilson IB, Ravazzola M, Orci L et al. (1986) Preproglucagon gene expression in pancreas and intestine diversifies at the level of post-translational processing. J Biol Chem, 261: 11880–11889. PubMed: 3528148. 3528148

[27] Ye Y, Keyes KT, Zhang C, Perez-Polo JR, Lin Y et al. (2010) The myocardial infarct size-limiting effect of sitagliptin is PKA-dependent, whereas the protective effect of pioglitazone is partially dependent on PKA. Am J Physiol Heart Circ Physiol, 298: H1454–H1465. doi:10.1152/ajpheart.00867.2009. PubMed: 20207816. 20207816

[28] Anagnostis P, Athyros VG, Adamidou F, Panagiotou A, Kita M et al. (2011) Glucagon-like peptide-1-based therapies and cardiovascular disease: looking beyond glycaemic control. Diabetes Obes Metab, 13: 302–312. doi:10.1111/j.1463-1326.2010.01345.x. PubMed: 21205117. 21205117

[29] Noyan-Ashraf MH, Momen MA, Ban K, Sadi A-M, Zhou Y-Q et al. (2009) GLP-1R agonist liraglutide activates cytoprotective pathways and improves outcomes after experimental myocardial infarction in mice. Diabetes, 58: 975–983. doi:10.2337/db08-1193. PubMed: 19151200. 19151200 PMC2661586

[30] Yan W, Zhang H, Liu P, Wang H, Liu J et al. (2013) Impaired mitochondrial biogenesis due to dysfunctional adiponectin-AMPK-PGC-1α signaling contributing to increased vulnerability in diabetic heart. Basic Res Cardiol, 108: 329. doi:10.1007/s00395-013-0329-1. PubMed: 23460046. 23460046

[31] Rajesh M, Mukhopadhyay P, Bátkai S, Patel V, Saito K et al. (2010) Cannabidiol attenuates cardiac dysfunction, oxidative stress, fibrosis, and inflammatory and cell death signaling pathways in diabetic cardiomyopathy. J Am Coll Cardiol, 56: 2115–2125. doi:10.1016/j.jacc.2010.07.033. PubMed: 21144973. 21144973 PMC3026637

[32] Ares-Carrasco S, Picatoste B, Benito-Martín A, Zubiri I, Sanz AB et al. (2009) Myocardial fibrosis and apoptosis, but not inflammation, are present in long-term experimental diabetes. Am J Physiol Heart Circ Physiol, 297: H2109–H2119. doi:10.1152/ajpheart.00157.2009. PubMed: 19820199. 19820199

[33] D’Souza A, Howarth FC, Yanni J, Dobryznski H, Boyett MR et al. (2011) Left Ventricle Structural Remodelling in the Prediabetic Goto-Kakizaki Rat. Exp Physiol 96(9): 875-888. PubMed: 21622965. 21622965 10.1113/expphysiol.2011.058271

[34] Lorell BH, Carabello BA ( Jul 252000) Left ventricular hypertrophy: pathogenesis, detection, and prognosis. Circulation Jul 25;102(4): 470-479. doi:10.1161/01.CIR.102.4.470. PubMed: 10908222. 10908222

[35] Regan TJ (1983) Congestive heart failure in the diabetic. Annu Rev Med, 34: 161–168. doi:10.1146/annurev.me.34.020183.001113. PubMed: 6344754. 6344754

[36] Lenski M, Kazakov A, Marx N, Böhm M, Laufs U (2011) Effects of DPP-4 inhibition on cardiac metabolism and function in mice. J Mol Cell Cardiol, 51: 906–918. doi:10.1016/j.yjmcc.2011.08.001. PubMed: 21871459. 21871459

[37] Rendell M, Drincic A, Andukuri R (2012) Alogliptin benzoate for the treatment of type 2 diabetes. Expert Opin Pharmacother, 13: 553–563. doi:10.1517/14656566.2012.656088. PubMed: 22296609. 22296609

[38] Rosenstock J, Sankoh S, List JF (2008) Glucose-lowering activity of the dipeptidyl peptidase-4 inhibitor saxagliptin in drug-naive patients with type 2 diabetes. Diabetes Obes Metab, 10: 376–386. doi:10.1111/j.1463-1326.2008.00876.x. PubMed: 18355324. 18355324

[39] Mellitzer G, Gradwohl G (2011) Enteroendocrine cells and lipid absorption. Curr Opin Lipidol, 22: 171–175. doi:10.1097/MOL.0b013e32834622a2. PubMed: 21464713. 21464713

[40] Cobble M (2012) Differentiating among incretin-based therapies in the management of patients with type 2 diabetes mellitus. Diabetology & Metabolic Syndrome, 4:8 10.1186/1758-5996-4-8 PMC3310739 22390369

[41] Sauvé M, Ban K, Momen MA, Zhou Y-Q, Henkelman RM et al. (2010) Genetic deletion or pharmacological inhibition of dipeptidyl peptidase-4 improves cardiovascular outcomes after myocardial infarction in mice. Diabetes, 59: 1063–1073. doi:10.2337/db09-0955. PubMed: 20097729. 20097729 PMC2844815

[42] Ishibashi Y, Matsui T, Ojima A, Nishino Y, Nakashima S et al. (2012) Glucagon-like peptide-1 inhibits angiotensin II-induced mesangial cell damage via protein kinase A. Microvasc Res;84(3): 395-398. doi:10.1016/j.mvr.2012.06.008. PubMed: 22750392. 22750392

[43] Mima A, Hiraoka-Yamomoto J, Li Q, Kitada M, Li C et al. (. 11 2012) Protective effects of GLP-1 on glomerular endothelium and its inhibition by PKCβ activation in diabetes. Diabetes. 11;61(11): 2967-2979. doi:10.2337/db11-1824. PubMed: 22826029. 22826029 PMC3478518

[44] Read PA, Khan FZ, Heck PM, Hoole SP, Dutka DP (2010) DPP-4 inhibition by sitagliptin improves the myocardial response to dobutamine stress and mitigates stunning in a pilot study of patients with coronary artery disease. Circ Cardiovasc Imaging, 3: 195–201. doi:10.1161/CIRCIMAGING.109.899377. PubMed: 20075143. 20075143

[45] Gros R, You X, Baggio LL, Kabir MG, Sadi AM et al. (2003) Cardiac function in mice lacking the glucagon-like peptide-1 receptor. Endocrinology, 144: 2242–2252. doi:10.1210/en.2003-0007. PubMed: 12746281. 12746281

[46] Tomas E, Habener JF (2010) Insulin-like actions of glucagon-like peptide-1: a dual receptor hypothesis. Trends Endocrinol Metab 21: 59–67. doi:10.1016/j.tem.2009.11.007. PubMed: 20018525. 20018525 PMC4085161

[47] Wang X-F, Zhang J-Y, Li L, Zhao X-Y (2011) Beneficial effects of metformin on primary cardiomyocytes via activation of adenosine monophosphate-activated protein kinase. Chin Med J (Engl), 124: 1876–1884. PubMed: 21740847. 21740847

[48] Xiao H, Ma X, Feng W, Fu Y, Lu Z et al. (2010) Metformin attenuates cardiac fibrosis by inhibiting the TGFbeta1-Smad3 signalling pathway. Cardiovasc Res, 87: 504–513. doi:10.1093/cvr/cvq066. PubMed: 20200042. 20200042

[49] Okerson T, Chilton RJ (2010) The Cardiovascular Effects of GLP-1 Receptor Agonists. Cardiovasc Ther. 10.1111/j.1755-5922.2010.00256.x PMC3488299 21167014

[50] Oeseburg H, De Boer RA, Buikema H, Van der Harst P, Van Gilst WH et al. (2010) Glucagon-like peptide 1 prevents reactive oxygen species-induced endothelial cell senescence through the activation of protein kinase A. Arterioscler Thromb Vasc Biol, 30: 1407–1414. doi:10.1161/ATVBAHA.110.206425. PubMed: 20448207. 20448207

[51] Burns KA, Vanden Heuvel JP (2007) Modulation of PPAR activity via phosphorylation. Biochim Biophys Acta, 1771: 952–960. doi:10.1016/j.bbalip.2007.04.018. PubMed: 17560826. 17560826 PMC2712836

[52] Wagner N, Jehl-Piétri C, Lopez P, Murdaca J, Giordano C et al. (2009) Peroxisome proliferator-activated receptor beta stimulation induces rapid cardiac growth and angiogenesis via direct activation of calcineurin. Cardiovasc Res, 83: 61–71. doi:10.1093/cvr/cvp106. PubMed: 19351742. 19351742

[53] Fan SC, Yu BC, Chen ZC, Chen LJ, Chung HH et al. (2010) The decreased expression of peroxisome proliferator-activated receptors delta (PPARdelta) is reversed by digoxin in the heart of diabetic rats. Horm Metab Res, 42: 637–642. doi:10.1055/s-0030-1253373. PubMed: 20446238. 20446238

[54] Kim HJ, Kim MY, Jin H, Kim HJ, Kang SS et al. (2009) Peroxisome proliferator-activated receptor {delta} regulates extracellular matrix and apoptosis of vascular smooth muscle cells through the activation of transforming growth factor-{beta}1/Smad3. Circ Res, 105: 16–24. doi:10.1161/CIRCRESAHA.108.189159. PubMed: 19461048. 19461048

[55] Piteau S, Olver A, Kim S-J, Winter K, Pospisilik JA et al. (2007) Reversal of islet GIP receptor down-regulation and resistance to GIP by reducing hyperglycemia in the Zucker rat. Biochem Biophys Res Commun, 362: 1007–1012. doi:10.1016/j.bbrc.2007.08.115. PubMed: 17803965. 17803965

