# Correction: Sitagliptin Reduces Cardiac Apoptosis, Hypertrophy and Fibrosis Primarily by Insulin-Dependent Mechanisms in Experimental type-II Diabetes. Potential Roles of GLP-1 Isoforms

**DOI:** 10.1371/annotation/df98874f-c1bd-4a13-b474-942fbb956287

**Published:** 2013-11-19

**Authors:** Belén Picatoste, Elisa Ramírez, Alicia Caro-Vadillo, Cristian Iborra, Jesús Egido, José Tuñón, Óscar Lorenzo

Dr. Sara Ares-Carrasco was not correctly included in the author byline. Dr. Ares-Carrasco should be listed as the fifth author and affiliated with Instituto de Investigaciones Sanitarias-Fundación Jiménez Díaz, Madrid, Spain. The contributions of this author are as follows: Performed the experiments. Dr. Ares-Carrasco's name should be abbreviated in the Author Contributions Statement as follows: SAC.

The correct Citation is: Picatoste B, Ramírez E, Caro-Vadillo A, Iborra C, Ares-Carrasco S, et al. (2013) Sitagliptin Reduces Cardiac Apoptosis, Hypertrophy and Fibrosis Primarily by Insulin-Dependent Mechanisms in Experimental type-II Diabetes. Potential Roles of GLP-1 Isoforms. PLoS ONE 8(10): e78330. doi:10.1371/journal.pone.0078330.

