# Correction: Sitagliptin Reduces Cardiac Apoptosis, Hypertrophy and Fibrosis Primarily by Insulin-Dependent Mechanisms in Experimental type-II Diabetes. Potential Roles of GLP-1 Isoforms

**DOI:** 10.1371/journal.pone.0091601

**Published:** 2014-02-28

**Authors:** The PLOS ONE Staff

There was an error in affiliation number 1 for the first, second, fourth, fifth, sixth, seventh and last authors. The correct affiliation is: IIS-Fundación Jiménez Díaz, Autónoma University, Madrid.

In addition, an affiliation for the last author was incorrectly omitted from the byline. In addition to institution number 1, Óscar Lorenzo is also affiliated with the following institution: Spanish Biomedical Research Centre in Diabetes and Associated Metabolic Disorders (CIBERDEM).
